# Primary diffuse large B-cell lymphoma of the central nervous system identified with CSF biomarkers

**DOI:** 10.1186/s12883-024-03761-6

**Published:** 2024-07-22

**Authors:** Valentin Loser, Amandine Segot, Laurence de Leval, Bettina Bisig, Jean-Philippe Brouland, Ekkehard Hewer, Carmen Barcena, Andreas F. Hottinger, Caroline Pot

**Affiliations:** 1 Department of Clinical Neurosciences, Service of Neurology, Lausanne University Hospital (CHUV)and, University of Lausanne , CHUV-BH07 Lausanne, Switzerland; 2 Department of Oncology, Service and Central Laboratory of Hematology, Lausanne University Hospital (CHUV)and, University of Lausanne , Lausanne, Switzerland; 3 Institute of Pathology, Department of Laboratory Medicine and Pathology, Lausanne University Hospital (CHUV)and, University of Lausanne , Lausanne, Switzerland; 4 Lundin Family Brain Tumor Research Centre, Departments of Oncology & Clinical Neurosciences, Lausanne University Hospital (CHUV)and, University of Lausanne , Lausanne, Switzerland

**Keywords:** PCNSL, Lymphoma, Cerebrospinal fluid, Biomarker

## Abstract

**Background:**

Diagnosis of primary diffuse large B-cell lymphoma of the central nervous system (PCNSL) is challenging and often delayed. MRI imaging, CSF cytology and flow cytometry have a low sensitivity and even brain biopsies can be misleading. We report three cases of PCNSL with various clinical presentation and radiological findings where the diagnosis was suggested by novel CSF biomarkers and subsequently confirmed by brain biopsy or autopsy.

Case presentations.

The first case is a 79-year-old man with severe neurocognitive dysfunction and static ataxia evolving over 5 months. Brain MRI revealed a nodular ventriculitis. An open brain biopsy was inconclusive. The second case is a 60-year-old woman with progressive sensory symptoms in all four limbs, evolving over 1 year. Brain and spinal MRI revealed asymmetric T2 hyperintensities of the corpus callosum, corona radiata and corticospinal tracts. The third case is a 72-year-old man recently diagnosed with primary vitreoretinal lymphoma of the right eye. A follow-up brain MRI performed 4 months after symptom onset revealed a T2 hyperintense fronto-sagittal lesion, with gadolinium uptake and perilesional edema. In all three cases, CSF flow cytometry and cytology were negative. Mutation analysis on the CSF (either by digital PCR or by next generation sequencing) identified the *MYD88* L265P hotspot mutation in all three cases. A B-cell clonality study, performed in case 1 and 2, identified a monoclonal rearrangement of the immunoglobulin light chain lambda (IGL) and kappa (IGK) gene. CSF CXCL-13 and IL-10 levels were high in all three cases, and IL-10/IL-6 ratio was high in two. Diagnosis of PCNSL was later confirmed by autopsy in case 1, and by brain biopsy in case 2 and 3.

**Conclusions:**

Taken together, 5 CSF biomarkers (IL-10, IL-10/IL-6 ratio, CXCL13, *MYD88* mutation and monoclonal IG gene rearrangements) were strongly indicative of a PCNSL. Using innovative CSF biomarkers can be sensitive and complementary to traditional CSF analysis and brain biopsy in the diagnosis of PCNSL, potentially allowing for earlier diagnosis and treatment.

## Background

Primary diffuse large B-cell lymphoma of central nervous system (PCNSL), also called primary large B-cell lymphoma of immune-privileged sites [1, 2], is a highly aggressive diffuse large B-cell lymphoma that is confined to the CNS, without any evidence of systemic lymphoma, and accounts for about 3–4% of all CNS tumors [3]. Diagnosis of PCNSL requires a high level of suspicion, as clinical and radiological presentation may be unusual. Definitive diagnosis is typically established by histopathological analysis of stereotactic or open brain biopsy (gold standard). When brain biopsy is not feasible or not contributory, the diagnosis becomes challenging. Several indirect ways may assist to establish the diagnosis: cerebrospinal fluid (CSF) cytology is the gold standard for diagnosing meningeal dissemination of PCNSL, but the sensitivity remains probably less than 16% [4]. CSF flow cytometry has been proposed to improve diagnosis accuracy in CNS localizations of hematologic malignancies [5], but its sensitivity in detecting PCNSL is between 3 and 23% [6–8]. To improve sensitivity with CSF cytology and flow cytometry, it is recommended to collect large CSF volume and perform multiple lumbar punctures. We report three cases of PCNSL with various clinical presentation and radiological findings where the diagnosis was suggested by novel CSF biomarkers and subsequently confirmed by brain biopsy or autopsy.

## Case presentation

### Case 1

A 79-year-old man presented with behavior disturbance, gait problems and anorexia with a loss of weight (10 kg), evolving over about 5 months. Neuropsychological examination revealed a multimodal disorientation, severe anterograde and retrograde amnesia, executive dysfunction, and non-lateralized attentional deficit. Neurological examination showed static ataxia with a tendency to retropulsion, and no other abnormalities.

**Fig. 1:**
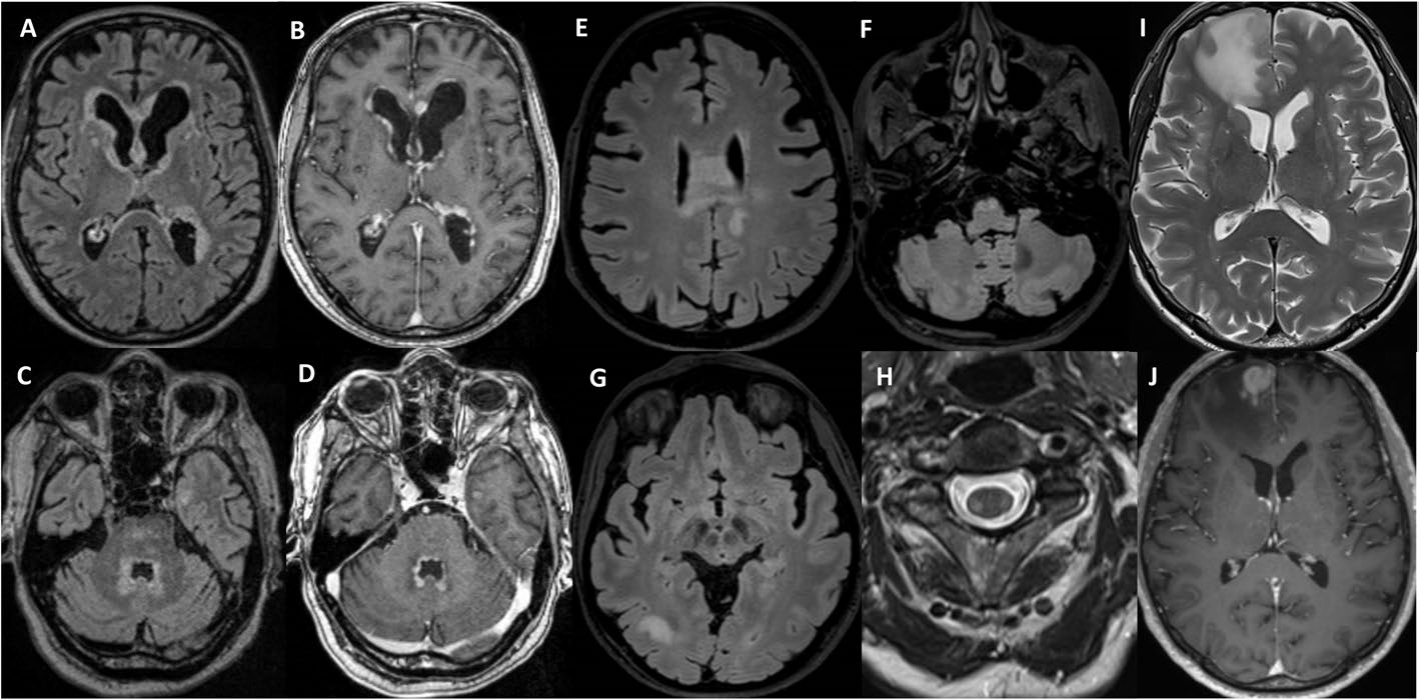
Brain MRI of the three cases. Case 1: Initial MRI with periventricular T2/FLAIR hyperintensities (**A**, **C**) and nodular gadolinium enhancement (**B**, **D**). Case 2: Brain MRI with T2/FLAIR hyperintensities of the corpus callosum extending to the corona radiata (**E**), of the right parieto-occipital subcortical white matter (**G**) and of the bilateral pyramids (**F**). Spinal MRI with bilateral lateral columns T2 hyperintensities (**H**). Case 3: Brain MRI with T2 hyperintense fronto-sagittal lesion and perilesional edema (**I**) and strong gadolinium enhancement (**J**)

A brain MRI 5 months after symptoms onset showed periventricular (adjacent to the lateral, third and fourth ventricles) T2-fluid-attenuated inversion recovery (FLAIR) hyperintensity, with partially nodular gadolinium enhancement (Fig. 1A-D). CSF analysis showed lymphocytic pleocytosis (194 cells/mm3, 98% lymphocytes), hyperproteinorachia (3881 mg/l), normal glucose and lactate and absence of intrathecal IgG synthesis.

Infectious causes were reasonably excluded. An extensive immunological workup was within normal limits, providing no evidence for a systemic disease, particularly a granulomatous vasculitis, sarcoidosis, nor IgG4-related disease. A salivary gland biopsy revealed a lympho-plasmocytic infiltrate without any granuloma or evidence of Sjögren disease. Whole-body CT scan and 18F-FDG PET were normal, and 3 CSF cytology and 4 CSF flow cytometry analyses were unremarkable. Slit-lamp ophthalmologic examination was normal. An open brain biopsy of a nodule adjacent to the left frontal horn, performed without prior glucocorticoid therapy, was not conclusive and only revealed a reactive astrogliosis and a slight lympho-histiocytic infiltrate.

At this point, the main suspicions were of an oncological disease of the CNS (particularly lymphoma), or an inflammatory granulomatous disease. As the neurological evolution was unfavorable, empiric treatment with IV methylprednisolone 1 g/day was introduced. After transient improvement, the patient’s neurological condition subsequently worsened with the onset of a focal status epilepticus requiring three lines of antiseizure medication, and a severe pneumonia. A second brain biopsy was considered, but because of the lesions being too deep, the risk–benefit ratio was judged unfavorable.

Targeted next generation sequencing (NGS) (using a custom capture-based panel covering 142 genes relevant for hematological neoplasms) was performed on DNA extracted from 4 pooled CSF samples and identified the *MYD88* L265P hotspot mutation at a variant allele frequency (VAF) of 6%, in addition to several other mutations. A B-cell clonality study identified a monoclonal rearrangement of the immunoglobulin light chain lambda (IGL*)* gene. An extensive CSF cytokine and chemokine panel analysis was performed. CSF CXCL-13 was 10′826 pg/ml, IL-10 level was 34 pg/ml and IL-10/IL-6 ratio was 0.48 (Table 1). Taken together, those results were strongly suggestive of a B-cell lymphoma. Unfortunately, the patient developed a pulmonary sepsis and passed away before treatment could be initiated.

**Table 1:** CSF diagnostic biomarkers in the 3 cases

	**IL-10 (pg/ml)**	**IL-10/IL-6 ratio**	**CXCL-13 (pg/ml)**	***MYD88* mutation analysis**^**b**^	**B-cell clonality assays**	**Cytology**	**Flow cytometry**
Case 1	34^a^	0.48	10′826^a^	L265P^a^	Monoclonal IGL gene rearrangement^a^	Neg (3x)	Neg (4x)
Case 2	52^a^	7.5^a^	3′053^a^	L265P^a^	Monoclonal IGK gene rearrangement^a^	Neg (1x)	Neg (1x)
Case 3	68^a^	16.2^a^	239^a^	L265P^a^	Not performed	Neg (1x)	Neg (1x)
Published cut-offs for PCNSL diagnosis	> 2.7–16.15 pg/ml (12)	> 0.72–1.6 (19–21)	> 80–90 pg/ml (9,10,13,14)				

*IL-10* Interleukin-10, *IL-6* Interleukin-6, *CXCL-13* Chemokine ligand 13, *MYD88* Myeloid differentiation primary response 88, *IGL* Immunoglobulin light chain lambda, *PCNSL* Primary central nervous system lymphoma, *Neg* Negative

^a^Values/test results suggestive of PCNSL

^b^Identification of the *MYD88* hotspot mutation, c.794 T > C p.(Leu265Pro) – most commonly referred to as L265P – according to reference transcript NM_002468.4. According to other reference transcripts this same mutation may be referred to as c.755 T > C p.(Leu252Pro) (NM_002468.5, corresponding to the currently approved MANE transcript), or as c.818 T > C p.(Leu273Pro) (NM_001172567.1 or LRG_157t1)

**Fig. 2:**
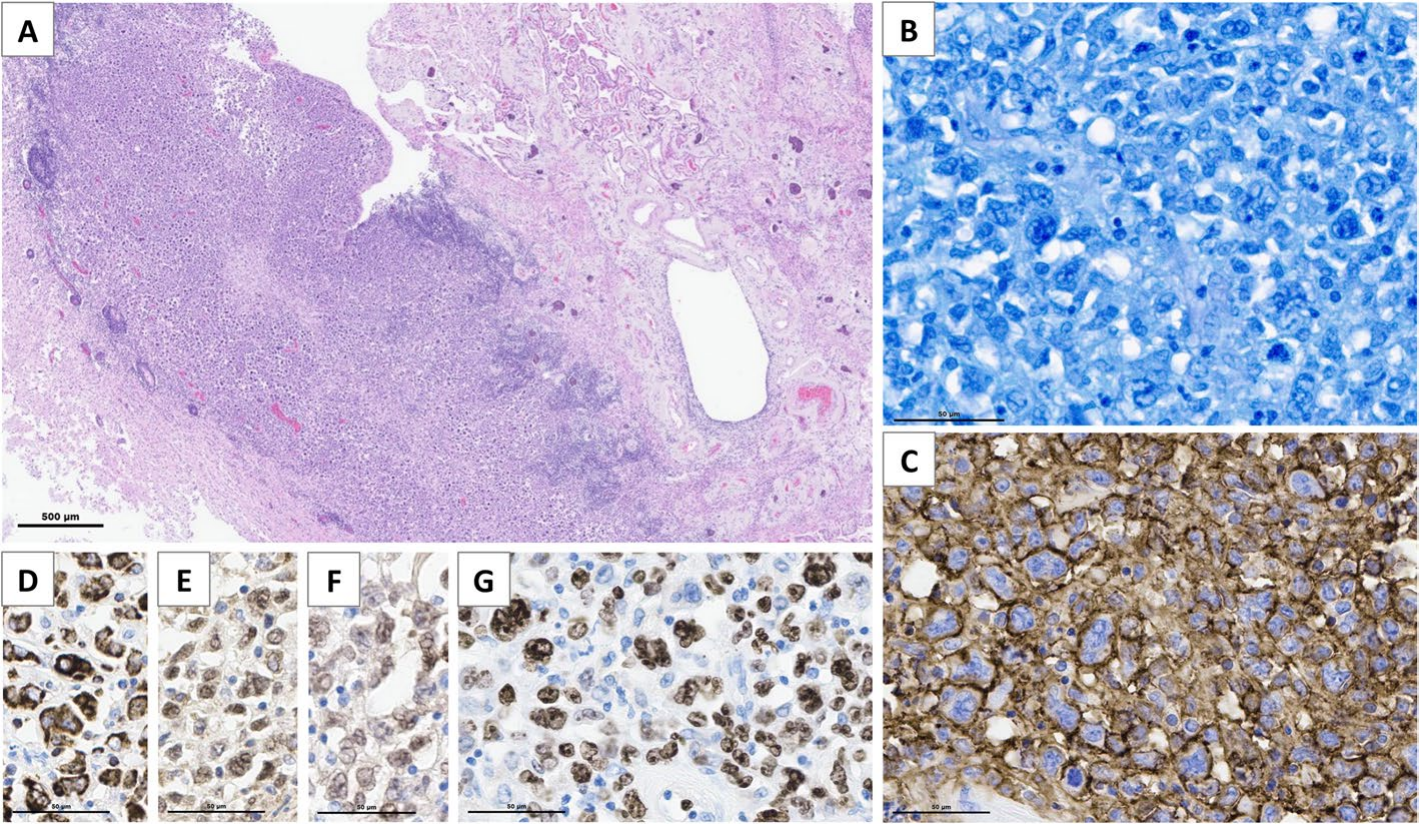
Brain autopsy findings in patient 1. Thickening of periventricular areas that are infiltrated by a diffuse population of large tumor cells without glandular or squamous differentiation (**A**, Hematoxylin–Eosin staining). The tumor is composed of a diffuse proliferation of large cells with a high nucleo-cytoplasmic ratio, mono- and multilobated nuclei, numerous mitoses (**B**, Giemsa staining) and high proliferation index Ki67/MIB1 near 80% (**G**). Tumor cells express CD20 (**C**), with coexpression of Bcl2 (**D**), Bcl6 (**E**) and MUM1 (**F**), and are monotypic for immunoglobulin light chain lambda, consistent with the diagnosis of primary diffuse large B-cell lymphoma of CNS (EBV negative, not shown)

Histological analysis of autopsied central nervous system tissue revealed a large B-cell lymphoma restricted to the CNS, CD5 + , lambda + , of non-germinal center-like phenotype, BCL2 + , MYC-, with strong proliferation (80%), EBV negative (Fig. 2). MYD88 mutation detection was not performed on autopsy tissue.

### Case 2

A 60-year-old woman presented with slowly progressive ascending sensory symptoms involving both feet, hands and trunk, associated with urinary symptoms, slight imbalance and dysarthria, evolving over about a year. A first brain and spinal MRI, performed 8 months after symptoms onset, was unremarkable. Neurological examination showed slight dysarthria, lower limbs tactile hypoesthesia with a T6 sensory level, increased reflexes in four limbs with a bilateral positive Hoffmann sign and toe flexor responses, and a sensory ataxia. A cervical or thoracic myelopathy was suspected.

Brain and spinal MRI performed 1 year after symptoms onset showed asymmetric T2-FLAIR hyperintensity lesions of the splenium of the corpus callosum, extending to the corona radiata, of the right subcortical parieto-occipital region and of the bilateral corticospinal tracts, starting from the pyramids, down to the lateral corticospinal bundles of the cervicothoracic medulla (Fig. 1E-H). There was no gadolinium enhancement. An MRI spectroscopy of the corpus callosum lesion was normal. CSF analysis showed no pleocytosis (2 cells/mm3), normal protein levels (386 mg/l), normal glucose and lactate and no intrathecal IgG synthesis.

Based on the MRI lesions, a leukodystrophy was initially suspected. A broad metabolic and genetic workup remained negative. However, the asymmetry of the brain lesions, the extent of the spinal cord involvement and the rapid onset of the lesions (within 3 months) strongly argued against a metabolic process. An extensive immunological workup was within normal limits, providing no evidence for a systemic disease. Whole-body CT scan was normal and 18F-FDG PET only showed a 18F-FDG hypermetabolism of the spinal cord lesions. One CSF cytology and flow cytometry analyses were within normal limits. Slit-lamp ophthalmologic examination was normal.

Targeted NGS on the CSF identified the *MYD88* L265P mutation at a VAF of 1.94%. A B-cell clonality study identified a monoclonal rearrangement of the immunoglobulin light chain kappa (IGK*)* gene. CSF CXCL-13 was 3053 pg/ml, IL-10 level was 52 pg/ml, and IL-10/IL-6 ratio was 7.5 (Table 1). Taken together, these findings were strongly suggestive of a B-cell lymphoma. An open brain biopsy of the right parieto-occipital lesion showed infiltration by a large B-cell lymphoma, CD5 + , kappa + , of non-germinal center-like phenotype, BCL2 + , MYC-, with strong proliferation (60%), EBV negative. Targeted NGS on tissue identified the *MYD88* L265P mutation at a VAF of 36%.

The patient was treated with MATRix chemotherapy (high-dose methotrexate, high-dose cytarabine, thiotepa and rituximab), followed by an intensification chemotherapy with carmustine-thiotepa and an autologous stem cell transplantation. The clinical and radiological response was initially favorable. Unfortunately, a radiological recurrence, confined to the CNS, occurred 4 months after the transplantation. A second-line chimeric antigen receptor (CAR) -T cell therapy with lisocabtagene maraleucel was started in April 2024.

### Case 3

A 72-year-old man was recently diagnosed with primary vitreoretinal lymphoma of the right eye, treated with intraocular dexamethasone and methotrexate. Diagnosis was made by vitreous humor cytopathology, which confirmed a CD20 + large B cell lymphoma. The first brain MRI performed at the time of the diagnosis was described as normal. The *MYD88* L265P mutation had already been demonstrated on that ocular sample, by pyrosequencing.

A follow-up MRI performed 4 months after diagnosis demonstrated a T2 hyperintense fronto-sagittal lesion, with strong gadolinium uptake and a surrounding perilesional edema (Fig. 1I-J). The patient showed no neurological symptoms at this stage, and neurological examination was normal. CSF analysis showed no pleocytosis (0 cells/mm3), normal protein levels (475 mg/l), normal glucose and lactate. One CSF cytology and flow cytometry were unremarkable.

DdPCR on the CSF identified the *MYD88* L265P mutation at a VAF of 0.56%. CSF CXCL-13 was 239 pg/ml, IL-10 level was 68 pg/ml, and IL-10/IL-6 ratio was 16.2 (Table 1). An open brain biopsy and surgical exeresis of the right frontal lesion confirmed a large B-cell lymphoma restricted to the CNS, CD5-, IgM kappa + , of non-germinal center-like phenotype, BCL2 + , MYC + , with strong proliferation (> 90%), EBV negative. Targeted NGS on tissue identified the *MYD88* L265P mutation at a VAF of 55%.

The patient was treated with PRIMAIN protocol chemotherapy (high-dose methotrexate, procarbazine and rituximab), followed by maintenance therapy with procarbazine. Six months later, he presented with a clinical and radiological recurrence, prompting second-line chemotherapy with TEDDi-R (ibrutinib, rituximab, temozolomide, etoposide, doxorubicine and dexamethasone). Two months later, a new recurrence occurred, prompting a third line of treatment with CAR-T cells (tisagenlecleucel) and stereotactic radiotherapy. Unfortunately, the patient rapidly developed a severe disorder of consciousness due to obstructive hydrocephalus and subsequently died in a palliative care institution.

## Discussion and conclusions

Those clinical cases illustrate the diagnostic challenges associated with PCNSL. Clinical presentation can be highly variable, the first case presenting as a nodular ventriculitis, mimicking a CNS neuro-inflammatory disease, the second one presenting clinically as a chronic myelopathy, with MRI lesions suggestive of leukodystrophy, and the third case presenting as a solitary brain nodule with perilesional edema and contrast enhancement. CSF cytology and flow cytometry yielded negative results. However, CSF IL-10 and CXCL13 level were above published cut-offs, which was strongly suggestive of PCNSL according to studies by Rubenstein et al. and Maeyama et al. [9, 10]. CSF IL-10/IL-6 ratio was negative in case 1 and positive in cases 2 and 3. Furthermore, the *MYD88* L265P hotspot mutation was found in all cases. Monoclonal rearrangement of the IGL gene was detected in case 1, and of the IGK gene was detected in case 2, while no B-cell clonality assay was performed in case 3. Final diagnosis was made on brain biopsy in case 2 and 3, but unfortunately was made only with autopsy in case 1.

### CSF cytokines/chemokines

CSF cytokines and chemokines are promising biomarkers of PCNSL but need to be validated [11, 12]. CXCL-13, IL-10 and IL-10/IL-6 ratio have been proposed to best correlate with PCNSL.

CXCL-13 is a B lymphocyte chemo attractant that binds to CXCR5 receptor and could contribute to lymphoma genesis [12]. Four recent studies demonstrated that an increase of CSF CXCL13 level had a sensitivity of 70–91% and specificity of 87–93% for CNS lymphoma diagnosis, with cutoffs between 80 and 90 pg/ml [9, 10, 13, 14]. According to a recent review from Li et al., CSF CXCL13 may also be a promising biomarker for prognosis assessment and disease monitoring in PCNSL [15]. However, CSF CXCL13 is not specific as it is known to be increased in other neurological diseases, such as neuroborreliosis or inflammatory disease of the CNS.

One of the most studied CSF cytokine biomarker is IL-10, a cytokine produced by type 2 T helper lymphocytes, monocytes, macrophages and B lymphocytes, whose immunosuppressive and growth factor functions may favor lymphoma genesis [16]. Its elevation was correlated with CNS lymphoma in at least nine studies, summarized in Nguyen-Them et al. paper, with a sensitivity of 59–96% and a specificity of 83–100% [12]. No clear cutoff value was admitted, varying from 2.7 to 16.15 pg/ml [12]. Concurrent elevation of CSF CXCL13 and IL-10 increased the diagnostic sensitivity to 97% and specificity to 97–99% according to two recent studies [9, 10]. As CSF IL-10 level may reflect tumor burden, it could also be a useful prognosis marker at diagnosis and in posttreatment evaluation [17, 18].

In addition, the elevation of CSF IL-10/IL-6 ratio was also correlated with PCNSL in three studies, with cut-offs above 0.72–1.6 harboring a sensitivity of 66–95.5% and a specificity of 91–100% [19–21].

### B-cell clonality assays and *MYD88* mutation

PCR-based analysis of immunoglobulin heavy and light chain gene rearrangements (B-cell clonality assays) can identify monoclonal B-cell populations in the CSF, with variable sensitivity according to studies [4, 22–24]. The rather low sensitivity may be due in part to the absence or the small number of circulating tumor cells in the CSF.

*MYD88* mutations, particularly the L265P hotspot mutation, are encountered in approximately half of PCNSL and, although not completely specific, are strongly suggestive of the diagnosis. The L265P mutation can be detected by various PCR or sequencing approaches, including ddPCR and targeted NGS panels. DdPCR is known for its superior sensitivity compared with other PCR techniques (such as real-time quantitative PCR) and NGS, as it requires only minimal amount of DNA. DdPCR has been shown to be an ideal technique for *MYD88* L265P molecular analysis in CSF sample [25]. *MYD88* L265P mutations were detected from CSF in 63.5–92% of PCNSL cases, with a specificity close to 100% [26–31]. Sporadic cases of *MYD88* L265P mutations in the CSF have been reported in other neurological diseases, such as multiple sclerosis [27]. Combined with an elevated CSF IL-10, sensitivity increased to 94% and specificity was 98% [27]. Combining *MYD88* L265P mutation detection and B-cell clonality assay on CSF cellular and cell-free DNA also improved the diagnosis of PCNSL [32].

Taken together, the combination of 5 CSF biomarkers (IL-10, IL-10/IL-6 ratio, CXCL13, *MYD88* mutation and monoclonal IG gene rearrangements) are strongly indicative of a PCNSL. Those cases illustrate that CSF analysis using innovative biomarkers can be a sensitive and complementary method to traditional CSF analysis (cytology and flow cytometry) and brain biopsy in the diagnosis of PCNSL. Moreover, the simultaneous use of multiple CSF biomarkers may enhance diagnostic performance, especially in patients that have an atypical clinical or radiological presentation. Multi-marker diagnostic algorithms, such as the one published by Maeyama et al., need to be further developed for patient with CNS lymphoma [10].

CSF biomarkers analysis has the advantage of being a minimally invasive and fast diagnostic method. It can be very useful for diagnosis in the absence of brain parenchymal lesion, when brain biopsy cannot be safely performed (e.g. in elderly/frail patients or when lesions are located in deep brain structures) and to overcome the challenge of sampling error with brain biopsy, especially due to prior glucocorticoid treatment [33]. Recently, it has been shown that targeted genotyping of CSF, especially for *MYD88* mutation detection, enables rapid diagnosis of CNS lymphoma and can accelerate the initiation of disease-directed treatment [34]. Methylation-based CSF liquid biopsies have also been shown to accurately classify the major three brain tumors (CNS lymphoma, glioblastoma and brain metastasis) [35]. Reviews on clinical application of liquid biopsy in CNS lymphoma was recently published by the RANO (Response Assessment in Neuro-Oncology) group [36]. However, histopathology provides additional information concerning the lymphoma “cell of origin” subtype (germinal center versus activated B-cell), the presence of rearrangements (MYC, BCL2 and BCL6) and the molecular subtype, informations that cannot currently be provided by non-invasive diagnostic method. Those informations can be of prognostic value, and molecular characteristics may lead, in the future, to the introduction of targeted treatment approaches.

## Conclusion

Diagnosis of PCNSL is laborious and is typically established by pathological evaluation of a brain biopsy. In cases where brain biopsy is infeasible or inconclusive, CSF analysis becomes a valuable diagnostic tool. Detection of neoplastic cells in the CSF by cytology and flow cytometry is a diagnostic alternative, but its sensitivity is very low. Innovative CSF diagnostic biomarkers exists, such as IL-10 and CXCL13 concentrations, IL-10/IL-6 ratio, B-cell clonality studies and *MYD88* mutation analysis, but they need to be validated in larger clinical studies and their use need to be better standardized, in order to improve the diagnosis and management of patient with PCNSL.

## Data Availability

No datasets were generated or analysed during the current study.

## Abbreviations

CSF: Cerebrospinal fluid
ddPCR: Droplet digital polymerase chain reaction
FLAIR: Fluid attenuation inversion recovery
IG: Immunoglobulin gene
IGK: Immunoglobulin light chain kappa gene
IGL: Immunoglobulin light chain lambda gene
NGS: Next generation sequencing
PCNSL: Primary central nervous system lymphoma
